# Role of Maternal Antibodies in Infants with Severe Diseases Related to Human Parechovirus Type 3^1^

**DOI:** 10.3201/eid2111.150267

**Published:** 2015-11

**Authors:** Yuta Aizawa, Kanako Watanabe, Tomohiro Oishi, Harunobu Hirano, Isao Hasegawa, Akihiko Saitoh

**Affiliations:** Niigata University Graduate School of Medical and Dental Sciences, Niigata, Japan (Y. Aizawa, T. Oishi, A. Saitoh);; Niigata University Graduate School of Health Sciences, Niigata (K. Watanabe);; Saiseikai Niigata Daini Hospital, Niigata (H. Hirano, I. Hasegawa);; University of California San Diego School of Medicine, San Diego, California, USA (A. Saitoh)

**Keywords:** Human parechovirus type 3, emerging infection, antibody, neonates, infants, intravenous immunoglobulin, maternal age, sepsis, meningoencephalitis, viruses

## Abstract

Human parechovirus type 3 (HPeV3) is an emerging pathogen that causes sepsis and meningoencephalitis in young infants. To test the hypothesis that maternal antibodies can protect this population, we measured neutralizing antibody titers (NATs) to HPeV3 and other genotypes (HPeV1 and HPeV6) in 175 cord blood samples in Japan. The seropositivity rate (>1:32) for HPeV3 was 61%, similar to that for the other genotypes, but decreased significantly as maternal age increased (p<0.001). Furthermore, during the 2014 HPeV3 epidemic, prospective measurement of NATs to HPeV3 in 45 patients with severe diseases caused by HPeV3 infection showed low NATs (<1:16) at onset and persistently high NATs (>1:512) until age 6 months. All intravenous immunoglobulin samples tested elicited high NATs to HPeV3. Our findings indicate that maternal antibodies to HPeV3 may help protect young infants from severe diseases related to HPeV3 and that antibody supplementation may benefit these patients.

## Abstract 2

Maternal antibodies may protect infants from severe illness caused by this pathogen.

Human parechoviruses (HPeVs) are small, nonenveloped, single-stranded, positive-sense RNA viruses classified in the genus *Parechovirus* of the family *Picornaviridae* (*1*). Among the 16 genotypes identified, the most common genotypes collected from patients are HPeV types 1 (HPeV1), 3 (HPeV3), and 6 (HPeV6) (*2*). HPeV type 4 is less common (*3*), and although HPeV type 5 has not been detected in Japan (*4*), it is circulating in the United States (*5*) and Europe (*6*). Generally, HPeV1 and HPeV6 cause mild gastroenteritis and respiratory infections in infants and children (*2**,**7*). HPeV1 (known as echovirus 22 until 1999) has occasionally been associated with severe diseases, such as encephalitis, acute flaccid paralysis, myocarditis, and neonatal sepsis (*2*), but in Japan, it has been isolated only from patients with mild diseases, including gastroenteritis, upper respiratory tract infection, and hand, foot, and mouth disease (*3*). In contrast, HPeV3, an emerging pathogen first reported in Japan in 2004 (*8*), causes severe diseases (e.g., sepsis and meningoencephalitis) in infants <3 months of age (*9*). HPeV3 infection can be accompanied by sepsis-like syndrome (*10**,**11*), which can lead to neurologic sequelae (*12*) and death (*13*), and has therefore attracted pediatricians’ attention (*14*). Clinical signs and symptoms and age of patients differ for various genotypes for reasons that are unclear.

We hypothesized that maternal antibodies may help protect neonates and young infants from severe diseases related to HPeV3. Seroepidemiologic data on HPeV3 have been available only from healthy persons or patients for whom viral infections other than HPeV3 have been diagnosed (*8**,**15*); data on perinatal antibody titers to HPeV3 have not been published. Although neutralizing antibody titers (NATs) have been reported as lower for mothers of infants with HPeV3 infection than for mothers of infants with other viral infections (*16*), little is known about patients infected with HPeV3 (*15*). Such data might help determine why severe diseases related to HPeV3 develop in some neonates and young infants. We measured NATs to HPeVs in cord blood from newborns and in serum samples from neonates and young infants with diseases related to HPeV3 infection who were admitted to hospitals in Niigata, Japan. In the latter group, samples were obtained from disease onset through convalescence. We also measured NATs in intravenous immunoglobulin (IVIG) preparations sold in Japan to determine if IVIG could be a potential treatment for HPeV3-related diseases in neonates and young infants.

## Materials and Methods

### Cord Blood Samples

Cord blood samples were collected during September 2013–January 2014 at Saiseikai Niigata Daini Hospital, a hospital affiliated with Niigata University in Niigata, Japan. Samples were collected from healthy newborns born at full term to women with uncomplicated pregnancies. Preterm infants (gestational age <37 weeks) were excluded because they have lower levels of maternally derived immunoglobulins than do full-term neonates (*17*). Also excluded were babies born to mothers with maternal conditions that could affect levels of maternal antibodies in newborns (e.g., pregnancy-induced hypertension and abnormalities in the placenta). After the umbilical cord was double-clamped and cut, an obstetrician obtained 3 mL of cord blood from the umbilical artery. The samples were centrifuged at 700 × *g* for 10 min at 4°C; plasma (>500 μL) was frozen at −80°C; and the samples were sent to the laboratory of the Department of Pediatrics, Niigata University, where they were stored at −80°C until analysis. The study was approved by the Ethics Committees of Niigata University and Saiseikai Niigata Daini Hospital. Informed written consent was obtained from the parents of all study participants.

### Patients with Severe Diseases Related to HPeV3

Since 2012, the viral causes of fever in neonates and young infants (i.e., <3 months of age) have been routinely evaluated by use of real-time PCR for enteroviruses (*18*), herpes simplex virus types 1 and 2 (*19*), and HPeVs (*20*) at Niigata University Hospital and its 40 affiliated hospitals in Niigata Prefecture (a population of ≈88,000 children <5 years of age). We evaluated patients with severe diseases related to HPeV3 who were admitted to Niigata University Hospital or 1 of its 11 affiliated hospitals during August 2013–September 2014. At onset, severe disease related to HPeV3 was defined as clinical symptoms and signs indicating sepsis or sepsis-like syndrome; disease status was confirmed by a positive result for HPeV3 by PCR analysis of samples of serum or cerebrospinal fluid (CSF). Information about patients’ contacts who were ill was obtained from interviews with parents and caregivers. With informed consent from parents, blood samples were collected prospectively at 3 and 6 months of age. Frozen serum samples were sent to the laboratory at Niigata University and stored at −20°C until analysis. The study was approved by the Ethics Committee of Niigata University.

### IVIG Preparations

Of the 5 commercially available IVIG preparations in Japan, 3 are from plasma pools of several thousand Japanese donors: polyethylene glycol (PEG)–treated IVIG (Venoglobulin IH; Mitsubishi Tanabe Pharma, Osaka, Japan), freeze-dried PEG-treated IVIG (Glovenin-I; Takeda, Osaka), and 2 batches of sulfonated IVIG (Venilon-I; Teijin Pharma, Tokyo, Japan); Another available IVIG preparation, ion-exchange resin-treated IVIG (Gammagard; Baxter, Tokyo), is from US donors; another, pH 4–treated IVIG (Sanglopor; CSL Behring, Tokyo), is from German donors. The plasma pools were collected during 2010–2012.

### Cell Line and Virus Cultivation

LLC-MK2 cells from the kidney of a healthy adult rhesus monkey were used for the neutralization assay (*3*). The cells were maintained in Eagle’s minimum essential medium (Sigma-Aldrich, St. Louis, MO, USA) containing 8% fetal bovine serum, 200 μg/mL gentamicin, and 2.5 μg/mL amphotericin B at 37°C, with 5% CO_2_, for 1 week before passage. HPeV1 Harris (*21*), HPeV3 A308/99 (*8*), and HPeV6 NII561–2000 (*3*) were cultured in LLC-MK2 cells to obtain a sufficient amount of working seed viruses. The viruses were stored at −80°C until the neutralization test. The median tissue-culture infectious dose (TCID_50_) was calculated by using the method of Reed and Muench (*22*).

### Neutralization Test

All samples (i.e., cord blood samples, serum samples from patients infected with HPeV3, and IVIG preparations) were subjected to neutralization testing. Serum samples for real-time PCR were used to measure NATs at disease onset. A total of 25 μL of 100 TCID_50_ viruses and 25 μL of serially diluted serum samples or IVIG preparation (started at 1:4 and then serially diluted twice until 1:2,048 in cord blood samples and IVIG preparations and until 1:512 in HPeV3-infected patients) were mixed in a 96-well plate and incubated at 37°C for 1 h. Then, 100 μL of suspended LLC-MK2 cells (10^6^/mL) was added to the wells. Cell controls and virus back titration were performed. The appearance of a cytopathic effect (CPE) was evaluated daily by light microscopy from day 3 and determined at day 10, when CPE was positive in control wells containing 100 TCID_50_ and was confirmed by virus back titration (*3*). CPE >50% was considered positive. The test was performed in triplicate when the IVIG preparations were evaluated.

### Real-time PCR

Real-time PCR was performed on serum or CSF samples collected from patients during the acute phase of the disease (<2 days after disease onset). CSF samples were centrifuged at 700 × *g* for 6 min at 4°C, and the supernatants were used in the analysis. Viral RNA was extracted from the serum and CSF samples by using a QIAamp MinElute Virus Spin Kit (QIAGEN, Valencia, CA, USA), according to the manufacturer’s instructions. Real-time reverse transcription PCR (RT-PCR) was performed by using a PrimeScript One Step RT-PCR Kit (TaKaRa, Tokyo, Japan) and the Thermal Cycler Dice Real Time System II (TaKaRa) with virus-specific primers and a TaqMan probe that targeted the conserved 5′ untranslated region (*20*). The thermocycling settings were 42°C for 5 min for cDNA synthesis; 95°C for 3 min followed by 45 cycles at 95°C for 5 s for denaturation; and 60°C for 40 s for annealing and extension.

### Genotyping of HPeVs

A seminested RT-PCR assay with modifications was used to amplify the viral protein (VP) 1 region (*23*). After viral RNA was converted to cDNA by using SuperScript VILO MasterMix (Invitrogen, Carlsbad, CA, USA), an HPeV-VP1S forward primer (5′-GGD ARR MTK GGD VAW GAY GC-3′) and HPeV-VP1AS2 reverse primer (5′-TCY ARY TGR TAY ACA YKS TCT CC-3′) pair was used in the first PCR; and HPeV-VP1AS (5′-CCA TAR TGY TTR TAR AAA CC-3′) was used as reverse primer in the second PCR. These primer sequences were determined by using the International Union of Pure and Applied Chemistry nucleotide ambiguity codes. The 20-μL PCR mixture contained 2 μL of cDNA, 0.5 μM of each primer, and 10 μL of iQ Supermix (Bio-Rad Laboratories, Hercules, CA, USA). The cycling conditions were 95°C for 3 min, followed by 30 cycles at 95°C for 15 s, 42°C for 30 s, and 72°C for 1 min, terminating in a final extension at 72°C for 10 min. Amplicon size was 830 bp. HPeV-positive samples were typed by sequencing the VP1 PCR product. When the amount of the second PCR product was insufficient to sequence the VP1 region, a third PCR was performed with the same primers used in the second PCR. The PCR products were purified by using Illustra ExoProStar (GE Healthcare, Tokyo, Japan) and sequenced with a BigDye Terminator v3.1 Cycle Sequencing Kit (Applied Biosystems, Foster City, CA, USA) on an automated sequencer (Applied Biosystems 3130xl Genetic Analyzer). Each type was determined by comparing the nucleotide sequence of the VP1 region with available HPeV sequences from the DNA data banks of DDBJ, EMBL, and GenBank.

Serum-derived cDNA was used for VP1 sequencing. When the serum sample was unsuccessful in amplifying the VP1 region, CSF or stool samples collected during the acute phase of disease were used. Stool samples were diluted with 1× phosphate buffered saline to 10% wt/vol suspensions, followed by vortex and centrifugation at 6,250 × *g* for 20 min at 4°C. The supernatants were filtered with a Millex-GV Filter Unit (EMD Millipore, Billerica, MA, USA) with a pore size of 0.22 μm. After sample preparation, stool samples underwent RNA extraction and cDNA synthesis reactions as described for previous samples.

### Statistical Analyses

All statistical analyses were performed by using SPSS Statistics 22.0 (IBM SPSS, Chicago, IL, USA). Geometric mean titers (GMTs) and seropositivity rates to HPeV1, 3, and 6 were compared by using the Kruskal-Wallis test and χ^2^ test, respectively. In the calculations, an antibody titer <1:4 was regarded as 1, and a titer >1:2,048 was regarded as 2,048. A 2-tailed p<0.05 was used to indicate statistical significance.

## Results

### NATs in Cord Blood Samples

**Figure 1:**
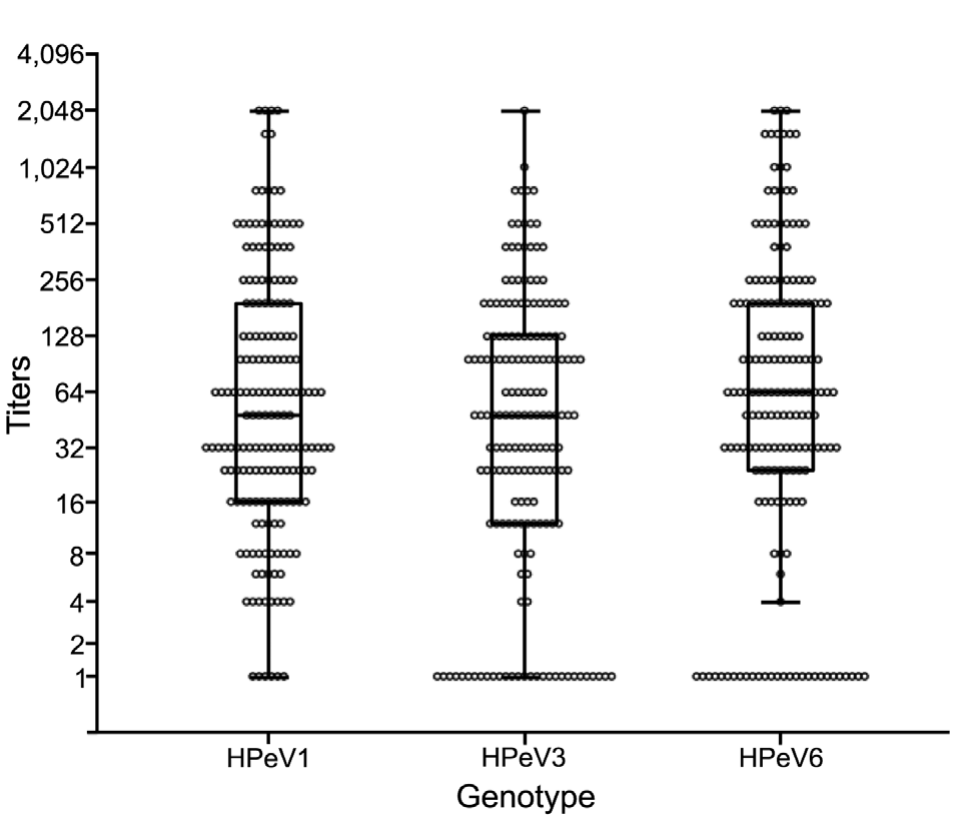
Distribution of neutralizing antibody titers to human parechovirus (HPeV) types 1, 3, and 6 in 175 cord blood samples from healthy neonates, Niigata, Japan, September 2013–January 2014. Titers are shown as reciprocal numbers. Boxes indicate first and third quartile values; bars within boxes indicate medians. Top and bottom bars indicate the 5th and 95th percentiles of data in a normal distribution. In the analysis, antibody titers <1:4 and >1:2,048 were regarded as 1 and 2,048, respectively.

During the study period, 175 cord blood samples were collected. Median gestation age was 39.7 (range 37.1–41.9) weeks, and median maternal age was 32 (range 16–44) years. GMTs of antibodies to HPeV1, 3, and 6 were 52.0 (95% CI 40.5–66.8), 33.9 (95% CI 25.4–45.3), and 48.9 (95% CI 35.7–66.9), respectively. GMTs showed no significant differences among the 3 genotypes (p = 0.17) (Figure 1).

### NATs in Patients with Severe Diseases Related to HPeV3

During the study period, 46 patients had a diagnosis of severe diseases related to HPeV, determined by PCR analysis of serum or CSF samples. NATs were measured in serum samples from 45 of these patients (1 patient was excluded because only a CSF sample was available on admission). Most (42/45 [93%]) were enrolled in the study during the epidemic of HPeV3 infection in Niigata in 2014. HPeV3 infection was identified in most (44/45 [98%]) HPeV-positive samples by sequencing the VP1 region of the virus by using cDNA derived from samples of serum (n = 40), CSF (n = 3), or stool cDNA (n = 1). The VP1 region could not be successfully amplified in 1 sample, so HPeV3 infection was diagnosed on the basis of a positive PCR result and a >4-fold increase in NATs to HPeV3.

The 45 patients with HPeV3-related diseases had a median age of 1 month (range 4 days–3 months, 21 days); 43 (96%) were <3 months of age, and 28 (62%) were male. For 25 (56%) patients, contact with a sick family member was identified. Clinical diagnoses at time of admission were sepsis (n = 37), sepsis-like illness (n = 7), and encephalitis with septic shock (n = 1).

All 45 serum samples at disease onset were available for neutralization testing. Most (42/45 [93%]) patients had a NAT of <1:4 to HPeV3 (HPeV3 negative). Three patients had low NATs to HPeV3: 1:4 in 1 (2%) patient and 1:16 in 2 (5%) patients.

**Figure 2:**
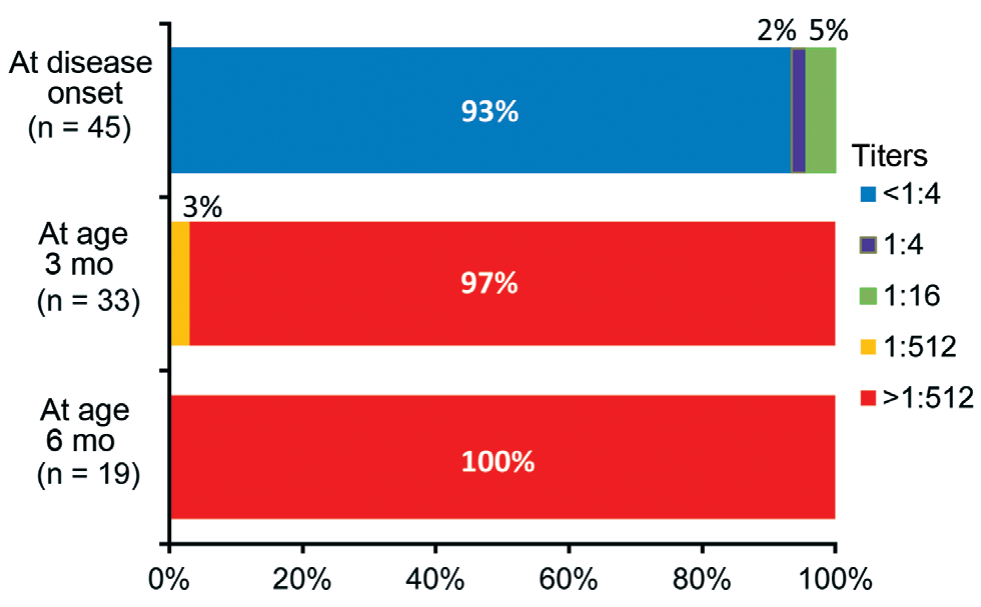
Change in neutralizing antibody titers to human parechovirus type 3 (HPeV3) in severe diseases related to HPeV3 in neonates and infants at disease onset and at 3 and 6 months of age, Niigata, Japan.

Follow-up NATs were measured in the 34 (76%) patients whose parents agreed to participate in the follow-up study. During ongoing follow-up, 33 patients were evaluated at age 3 months and 19 patients at age 6 months. NATs to HPeV3 were >1:512 in all patients at age 3 months and remained >1:512 at age 6 months (Figure 2).

### HPeV Seropositivity in Cord Blood Samples

Because the maximum antibody titer at disease onset was 1:16 in patients infected with HPeV3 and animal studies have shown high protection against enteroviral infection at titers of 1:32 (*24*), we chose a titer of >1:32 to indicate the level of neutralizing antibody necessary to prevent infection. At this level, seropositivity rates in cord blood samples for HPeV1, 3, and 6 were 65%, 61%, and 71%, respectively, and did not significantly differ among the 3 genotypes (p = 0.12) (Table 1). When the data were stratified by maternal age, the seropositivity rate for HPeV3 declined as maternal age increased (Table 1). When mothers were divided into age groups of 16–24 years (n = 11), 25–34 years (n = 107), and 35–44 years (n = 57), the group of youngest mothers had significantly higher GMTs (336.5, 95% CI 176.1–642.9) than those for older mothers (31.9 [95% CI 22.0–46.4] for mothers ages 25–34 and 24.4 [95% CI 15.4–38.6] for mothers ages 35–44; p<0.001). In contrast, more cord blood samples had a titer <1:4 for HPeV3 (n = 29, 17%) than for HPeV1 (n = 6, 3%), and seropositivity rates for these 2 groups differed significantly when a lower cutoff value (1:4) was considered (83% vs. 97%, respectively; p<0.001).

**Table 1:** Numbers and rates of seropositivity to HPeV types 1, 3, and 6 in cord blood samples from healthy full-term infants, by maternal age, Niigata, Japan, September 2013–January 2014*

HPeV type	Maternal age, no. (%) samples			
All ages, n = 175†	16–24 y, n = 11	25–34 y, n = 107	35–44 y, n = 57
HPeV1	113 (65)	6 (55)	75 (70)	32 (56)
HPeV3	107 (61)	11 (100)	68 (64)	28 (49)
HPeV6	125 (71)	8 (73)	69 (64)	48 (84)

*HPeV, human parechovirus. A cutoff of 1:32 was used for HPeV1, 3, and 6. †Antibody positivity rates compared among the 3 genotypes; p = 0.12.

### NATs in IVIG Preparations

All IVIG preparations we examined contained high NATs to all HPeVs (Table 2). NAT was ≥1:1,024 to HPeV1 and HPeV6 and ≥1:512 to HPeV3. No differences in antibody levels were observed among IVIG preparations originating from Japan, the United States, and Germany.

**Table 2:** NATs to HPeV types 1, 3, and 6 in intravenous immunoglobulin preparations commercially available in Japan*

Preparation	Country†	Year obtained	IVIG treatment	NATs		
				HPeV1	HPeV3	HPeV6
Batch 1	Japan	2010, 2011	PEG	1:1,024	1:512	1:2,048
Batch 2	Japan	2010	Freeze-dried PEG	1:1,024	1:512	1:1,024
Batch 3-1	Japan	2011	Sulfonation	1:1,024	1:512	1:2,048
Batch 3-2	Japan	2011, 2012	Sulfonation	1:1,024	1:1,024	1:2,048
Batch 4	United States	2012	Ion-exchange resin	1:1,024	1:512	1:2,048
Batch 5	Germany	2012	pH4	1:2,048	1:512	1:2,048

*HPeV1, human parechovirus type 1; IVIG, intravenous immunoglobulin; NATs, neutralizing antibody titers; PEG, polyethylene glycol.  †Country where IVIG was obtained.

## Discussion

Our findings show that NATs to HPeV3 were low (<1:32) in ≈40% of cord blood samples from healthy neonates, so this population could be at risk for developing severe diseases related to HPeV3. In addition, negative or low NATs (<1:16) were observed in all HPeV3-infected patients at disease onset. During follow-up, these titers were elevated and remained high (>1:512) until patients were 6 months of age. These data strongly suggest that maternal antibodies help protect neonates and young infants from severe disease related to HPeV3.

When the data were stratified by maternal age, the proportion of samples that were seropositive for HPeV3 decreased as maternal age increased. This finding suggests that neonates and young infants born to older mothers might be more likely to develop severe diseases related to HPeV3. One possibility is that NATs to HPeV3 wane if no reinfection or boosting of HPeV3 occurs in mothers. However, we found no significant differences in NATs and seropositivity rates to HPeV1, 3, and 6 at a cutoff of 1:32. Thus, for reasons yet to be identified, HPeV3 appears to be more pathogenic than HPeV1 and HPeV6 in neonates and young infants. When we used a cutoff of 1:4, seropositivity rates for HPeV3 and HPeV1 were significantly different (p<0.001). The different seropositivity rates at a lower cutoff might explain the differences in age distribution of children infected with HPeV1 compared with those infected with HPeV3, although no difference in seropositivity rates was found between HPeV3 (83%) and HPeV6 (84%) (p = 0.885).

We used LLC-MK2 cells for the viral assay of all HPeVs, including HPeV3, because we previously found that HPeV3 efficiently replicates in this cell line (*3*). HPeV3 seropositivity rates and the method of the neutralization assay were compared with those in previous reports (Table 3). A study in Japan’s Aichi Prefecture (≈200 miles from Niigata) reported a HPeV3 seropositivity rate of 74% among 92 persons 15–39 years of age (*8*). Our results are consistent with these findings, although Vero cells were used in the previous neutralization test and the cutoff was set arbitrarily at 1:8. In contrast, a study found low seropositivity rates for HPeV3 (FI0688, a strain isolated from the stool sample of a healthy child in Finland) among adults in Finland (13%; n = 72) and the Netherlands (10%; n = 77) (*15*). Vero cells were also used in those neutralization assays, and the cutoff was set arbitrarily at 1:16. The difference found in these reports (*8**,**15*) might result from the different virus strains used in the neutralization tests. HPeV3–150237 (the clinical isolate in the Netherlands) was not neutralized by anti-HPeV3 A308/1999 (the first reported isolate from a fecal sample of a 1-year-old girl with transient paralysis in Japan) (*8*) antibody in vitro (*25*). Although the populations studied differ geographically, location might have no effect because HPeV3 circulates worldwide and HPeV3 strains in Japan and Europe are closely related (*26*).

**Table 3:** Summary of rates of HPeV type 3 seropositivity and neutralization assay methods used in previous studies and in study in Niigata, Japan*

Study location	Seropositivity rate, % (no. samples)	Maternal age range, y	Cutoff	Cells used for viral culture	Virus strain
Aichi, Japan (*8*)	74 (92)	15–39	1:8	Vero	A308/1999
Finland (*15*)	13 (72)	21–40	1:16	Vero	FI0688
Netherlands (*15*)	10 (77)	16–60	1:16	Vero	FI0688
This study	61 (175)†	16–44	1:32	LLC-MK2	A308/1999

*HPeV, human parechovirus. †Maternal antibody seropositivity rate.

The study in Finland and the Netherlands described antibody response after HPeV3 infection in 3 children whose individual responses varied: no response, a decrease after temporary elevation, and sustained elevation (*15*). In contrast, NATs were elevated and remained high after HPeV1 (*27*) and HPeV6 infection (*15*). However, the report from Finland and the Netherlands used isolation of virus from stool samples to diagnose HPeV3 infection, without confirmation of clinical manifestations. HPeV3 is detectable in stool samples of healthy children (*28*); therefore, the 3 patterns mentioned in that study might include such cases. To avoid confounding infection and virus detection, HPeV3 infection should be clinically suspected and then confirmed by PCR analysis of sterile samples (e.g., serum or CSF). A strength of our study is that HPeV3 infection was confirmed in all patients by PCR analysis of serum or CSF samples. Also, low NATs at disease onset were later elevated and remained high during convalescence of patients with HPeV3-related diseases. This pattern has also been observed in infections with viruses closely related to HPeVs, such as enteroviruses (*29*).

Our findings of high NATs to HPeV1, 3, and 6 in all IVIG preparations are consistent with the seropositivity rates in cord blood samples. In contrast, the NATs to HPeV3 contained in Dutch IVIG preparations were low (1:10–1:40), although NATs to HPeV1 were similar (1:1,280–1:2,560) (*25*). In that study, HPeV3–150237 and HPeV3 A308/1999 were used in the neutralization assay with Vero cells. However, we used HPeV3 A308/1999 with LLC-MK2 cells. The use of a different cell line in the neutralization assay might affect results.

A standard method should be established for the neutralization assay for HPeV3. Previous studies used Vero cells for HPeV3 and HT29 cells for HPeV1 and HPeV6 (*15**,**25*) because of differences in cell tropism in the laboratory. Ideally, the same cell line should be used for assays for different HPeVs. Our findings indicate that using LLC-MK2 cells might be preferable in a neutralization assay for HPeVs because all 3 HPeVs replicate efficiently in LLC-MK2 cells and effects of using different cell lines can be excluded when interpreting results.

HPeV3 infection can cause severe diseases in neonates and young infants and may require intensive care and ventilator support. Patients with severe illness may need additional therapy because no current therapy is effective for HPeV-related diseases. One report described successful treatment with IVIG therapy for a 5-month-old boy with HPeV1 infection who developed severe myocarditis and dilated cardiomyopathy (*30*). Antibody titers increased after infection, and the IVIG contained high NATs. Currently, no data are available on the effectiveness of IVIG treatment for HPeV3 infection. In neonates with severe enteroviral infection, administration of IVIG containing NATs to the corresponding serotype resulted in rapid resolution of viremia and viruria because such patients have low NATs to the specific enteroviral serotype (*31*). Our findings suggest that IVIG treatment may be beneficial for patients with HPeV3 infection because these patients have low NATs, and IVIG preparations that are commercially available in Japan contain high NATs to HPeV3 in vitro. These high titers may help neutralize HPeV3 when used at an appropriate time during the course of infection. Future studies should investigate the efficacy of IVIG treatment for patients with HPeV3 infection, especially for patients with severe disease requiring intensive care or mechanical ventilation.

This study has some limitations. First, because cord blood samples were collected in only 1 hospital during the study period, GMT and HPeV seropositivity rates might not be generalizable to other regions of Japan or to other countries. Second, we used LLC-MK2 cells because of our previous favorable experience in isolating HPeV3 from clinical samples (*3*), and we chose HPeV3 A308/1999 because it is an HPeV3 reference strain (*2*). However, other cell lines and virus strains may need to be included in the system to evaluate differences in cell lines and viral strains. Third, we were unable to evaluate NATs of mother–infant pairs with or without HPeV3 infection. Maternal sampling with a control group may further clarify the contribution of maternally derived antibodies to severe disease related to HPeV3. Finally, follow-up samples from infants at ages 3 months and 6 months were collected from only some of the study participants because the study is ongoing.

We have shown a correlation between low titers of maternally derived antibodies to HPeV3 and development of severe diseases related to HPeV3 infection in neonates and young infants. Commercially available IVIG in Japan contains high titers to HPeV3 and thus might be an option for preventing or treating severe HPeV3-related diseases in this population.
